# Nutritional Quality of Plant-Based Fish and Seafood Analogs: A Study of the Italian Market

**DOI:** 10.3390/foods14030394

**Published:** 2025-01-25

**Authors:** Lara Chehade, Donato Angelino, Cristian Del Bo’, Rebecca Maggioni, Nicoletta Pellegrini, Patrizia Riso, Daniela Martini

**Affiliations:** 1 Department of Food, Environmental and Nutritional Sciences (DeFENS), Division of Human Nutrition, Università degli Studi di Milano, 20133 Milano, Italy; 2 Department of Bioscience and Technology for Food, Agriculture and Environment, University of Teramo, 64100 Teramo, Italy; 3 Department of Agricultural, Food, Environmental and Animal Sciences, University of Udine, 33100 Udine, Italy

**Keywords:** fish analogs, food labeling, plant-based fish, Nutri-Score, nutritional quality

## Abstract

Among plant-based analogs, fish and seafood analogs (PBFSAs) represent a growing sector. This study analyzed the nutritional quality of PBFSAs in Italy and compared it to their animal-based counterparts. Nutritional declarations, ingredient lists, and claims were collected from PBFSA food labeling. Nutri-Scores of PBSFAs and animal-based counterparts were also determined as nutritional quality indicators. Fifty-one products were collected, with most attributed to tuna, salmon, and cod categories (n = 18, 12, and 14, respectively). Results showed large heterogeneity in nutritional quality, with cod products having higher energy (217 (201–257) kcal/100 g), protein (10.5 (7.9–13.0) g/100 g), and carbohydrate (19.4 (14.2–26.0) g/100 g) levels, while tuna and salmon products had a higher fat content (15.0 (10.0–19.7) and 13.5 (5.0–17.0) g/100 g, respectively). Products with fiber or fat nutrition claims did not necessarily indicate higher fiber or lower fat content, while products with a protein claim had a higher protein content. Most animal-based counterparts, except cod and sturgeon caviar, received an “A” Nutri-Score, and often scored better than the PBSFA due to lower salt content. In conclusion, PBFSAs on the market should not be considered animal product analogs regarding nutritional quality, but drawing definitive conclusions is challenging due to the limited number and high variability of the products. However, these findings provide insights that may improve PBFSA nutritional quality, such as decreasing salt and sugar content, for people trying to incorporate such foods into their diet.

## 1. Introduction

Unsustainable food systems not only affect our health but also that of our planet. There have been growing concerns regarding current practices of food production, processing, transport, distribution, preparation, and consumption, and their environmental impact. This may be largely due to the fact that more than one-third of all human-caused greenhouse gas (GHG) emissions are linked to food systems [1]. Additionally, a significant global increase in food production was noted in the last 50 years, particularly in animal-derived foods (ADFs). This increase reached up to 403% for meat and meat products, 165% for milk and milk products, 355% for fish and seafood, and 513% for eggs [2]. Such an increase in production poses a threat to the sustainability of our food systems as it plays a major role in environmental degradation, including excessive GHG emissions, water use, and land use.

To address these issues, a dietary shift towards plant-based diets should be considered. Research has shown that adopting a diet higher in plant-based foods has the potential to mitigate environmental impacts. The shift can decrease diet-related GHG emissions by 49%, land use by 76%, and blue and green water use by 14% and 21%, respectively [3,4]. Healthwise, diets predominantly consisting of plant-based foods and low in salt, saturates, and added sugars have been associated with a decreased risk of all-cause mortality and non-communicable diseases (NCDs) [4,5].

For all these reasons, it is urgent to find strategies for promoting a shift towards sustainable healthy diets that are high in plant-based foods and moderate in animal-based foods. Of course, this transition can be achieved by the use and consumption of fresh/raw foods, such as fresh fruits and vegetables, and both fresh and dried legumes. However, taking into account the characteristics of modern life—often marked by fast-paced routines, limited time for grocery shopping, and minimal cooking skills—and the appeal to consumers who value familiar characteristics of meat or fish, the food industry has responded by introducing a wide range of plant-based products that mimic the appearance, taste, and texture of animal-based foods [6,7,8].

While most concerns are linked to the high consumption of land animal products, such as the meat, dairy, and eggs obtained from cows, pigs, and poultry, fish has also garnered attention. The consumption of fish has been acknowledged by international organizations such as the Food and Agriculture Organization (FAO) and the World Health Organization (WHO) thanks to its great nutritional value [9]. It is a rich source of nutrients that are essential for metabolic and hormonal functions. Among these nutrients are iodine, selenium, vitamin D, and proteins [10]. Furthermore, fish and seafood are among the richest sources of omega-3 fatty acids, such as eicosapentaenoic acid (EPA) and docosahexaenoic acid (DHA), which have been shown to have cardiovascular benefits and support overall well-being [11,12]. Despite its great nutritional value, the consumption of fish has been associated with environmental issues, especially related to overfishing. Indeed, it has been estimated that approximately 62% of world fish populations are being harvested at their maximum sustainable levels and 37% are being overfished [13]. There also are health concerns associated with eating certain types of fish and shellfish, particularly regarding the possible presence of mercury and other toxic heavy metals due to the pollution of sea water [14]. Additionally, some people experience allergies triggered by various seafood products [15].

These factors push towards finding alternatives to seafood consumption. One specific approach involves the use of plant-based fish and seafood analogs (PBFSAs), which imitate the taste, consistency, and appearance of their animal counterparts [16]. The PBFSA category makes up only a small fraction of the plant-based alternatives market [17], but it is seeing rapid growth. Their global market was valued in the millions of dollars in 2023, and analysts expect it to continue expanding significantly over the next 10 years [18,19,20,21]. In fact, Europe continues to invest in the research and development of these products, with key initiatives including the European Union’s €2 million “Seafood Alg-ternative” project, and Denmark’s and France’s investment in projects like Umiami’s facility [21]. Despite their growing market, current PBFSAs are facing challenges in Italy, such as a general lack of sensory appeal, that may limit their acceptance [22]. In addition to that, their nutritional quality has not yet been thoroughly analyzed. Therefore, understanding the actual nutritional quality and composition of these products is important. While previous research has evaluated the nutritional quality of PBFSAs launched across the global market, to the best of our knowledge, this is the first study to nutritionally evaluate what is currently available to consumers in the Italian market, providing insights into products that people in Italy can currently purchase and consume, while also highlighting critical nutrients. Because of that, and in an effort to support the urgent need to transition towards evidence-based healthy and sustainable diets, our study aims to fulfil the following three objectives: (i) examine the nutritional profiles of PBFSAs that are commercially sold in Italy by gathering nutritional information listed on their packaging, (ii) perform a nutritional comparison between the PBFSAs with their fish-based counterparts, and (iii) calculate the Nutri-Score for both the PBFSAs and fish-based products to assess their overall nutritional quality.

## 2. Materials and Methods

### 2.1. Product Selection

Product selection was performed according to a protocol described elsewhere [23]. All products included in this study are sold in retail grocery stores in Italy. These stores include the following: Bennet Drive, Carrefour, Conad, Coop, Crai, Decò, Esselunga, Il Gigante, Iper, Tigros, and Unes. Some stores known for selling specialized products were also added to the list: Amorum, Bio-Salute, Coccole di Gusto, Cortilia, Cuore Vegano Shop, iVegan, Naturitas, and Vegano Bio. To ensure consistency among the products obtained, information was collected from the websites of these stores between March 2024 and May 2024, only if the product was available online for purchase in at least one shop. A product was excluded if any side of the packaging was incomplete or not presented, or if the images of the nutrition labeling, declaration, and ingredient list were unclear.

### 2.2. Data Extraction and Nutri-Score Calculation

Product name, weight, ingredients, and nutritional values including the energy (kJ/100 g and kcal/100 g), total fat (g/100 g), saturates (g/100 g), total carbohydrates (g/100 g), sugars (g/100 g), fiber (g/100 g), protein (g/100 g), and salt (g/100 g) of each PBFSA were extracted and reported in line with the Council Regulation (EU) n. 1169/2011 [24].

Additionally, data on the presence of declarations regarding nutrition claims for energy, fat, protein, fiber, salt, and vitamins and minerals, in line with the Council Regulation (EC) No 1924/2006 [25], were gathered. Data on organic declarations, in line with the Council Regulation (EC) No 834/2007 [26], and those on gluten-free declarations, in line with the Council Regulation (EC) No 828/2014 [27], were also collected.

The extracted data were cross-validated by comparing them on the different websites of the retailers selling the products or directly on the website of the manufacturers. The accuracy of the extracted data was verified by two authors (R.M. and D.M.). Discrepancies were discussed and reviewed by a third reviewer (L.C.).

The products were classified into groups based on the following criteria: the category of fish or seafood they aim to replicate, whether they had an organic declaration, whether they had at least one nutrition claim, and whether it was indicated that they were gluten-free. Based on the main fish or seafood descriptive names found on the food labeling of the PBFSA, the products were grouped into the following 7 major food categories: tuna, salmon, cod, mackerel, squid, shellfish, and roe. The plant-based alternatives were compared against the following corresponding fish counterparts to evaluate nutritional quality: tuna in oil (drained), fresh salmon, cod fish sticks, mackerel fillet in oil, frozen squid, prawns, and sturgeon caviar. The nutritional values of the animal-based counterparts were collected from the Food Composition Database for Epidemiological Studies in Italy (BDA) [28] (Supplementary Table S1). To ensure comparability, the counterparts were selected based on (1) the available products in the BDA and (2) their similarity to the most common PBFSA products in each PBFSA category in terms of use and preparation methods (e.g., in oil, breaded).

A Nutri-Score, which is a simplified label using letters and colors to help indicate the nutritional quality of a product, was calculated for all the plant-based and animal-based products included in this study. Briefly, positive points were assigned to positive components (protein, fiber), and negative points to components that should be limited (energy, sugars, saturates, and salt), with salt contributing up to 20 points, all per 100 g of the product. Then, the total score was determined by subtracting the total negative points from the positive points. This score was then converted into a color (dark green to dark orange) and a letter (A to E) using specific cutoffs. The calculation was done in agreement with the algorithm described elsewhere [29].

### 2.3. Statistical Analysis

The statistical analysis was performed with the Statistical Package for Social Sciences software (IBM SPSS Statistics, Version 26.0, IBM Corp., Chicago, IL, USA). The significance level was set at *p* < 0.05.

Data are expressed as medians and interquartile ranges, as the Kolmogorov–Smirnov test rejected the normality distribution of the data. Differences among PBFSA categories in terms of energy, macronutrients, fiber, and salt were assessed by means of the Kruskal–Wallis test with multiple pairwise comparisons to determine specific group differences. Additionally, the Mann–Whitney test was used to examine the differences in terms of energy, macronutrients, fiber, and salt between variables belonging to products with or without nutrition claims, organic declarations, and gluten-free indications.

## 3. Results

Table 1 shows the number and overall characteristics of the PBFSAs. A total number of 51 PBFSAs were included in this study. The product distribution across the seven categories was as follows: tuna (35.3%), salmon (23.5%), cod (27.5%), mackerel (2.0%), squid (2.0%), shellfish (7.8%), and roe (2.0%). There was only one product for each of the plant-based mackerel, squid, and roe categories. More than half of the total products (51%) had at least one nutrition claim, 7.8% had organic declarations, and 9.8% of the products had gluten-free indications. The most common nutrition claims shown on PBFSAs were ones related to protein, fat, and fiber. Of the products having at least one nutrition claim, only three products referred to vitamins and minerals, all specifying vitamin B12, and one of them also mentioned iron.

Regarding formulations, it was noted that 69% of the products were formulated mainly from a mixture of cereals and legumes, 19% from cereals only, 10% from legumes only, and 2% from algae.

### 3.1. Nutritional Composition of PBFSAs

Energy, macronutrient, fiber, and salt contents of the different PBFSA categories are shown in Table 2.

When comparing the nutritional content among the different PBFSA categories, some notable differences were observed. Cod had a significantly higher energy and protein content than shellfish, as well as significantly higher carbohydrate levels than tuna (*p* < 0.01) and salmon (*p* < 0.05). Interestingly, there were no significant differences among the PBFSA categories in terms of sugars, fiber, and saturates. However, tuna exhibited significantly higher total fat content compared to shellfish. Regarding salt content, only salmon had significantly higher levels than cod.

### 3.2. Nutrition Claims, Organic Declarations, and Gluten-Free Indications

Table 3 presents the nutritional content of PBFSAs, categorized by the presence of nutrition claims, including fat, protein, fiber, and organic declarations, as well as gluten-free indications. Two nutrition claims were related to vitamin B12.

Products with at least one nutrition claim were significantly higher in protein (*p* < 0.05) and lower in salt (*p* < 0.01) than products without a nutrition claim. As expected, there was roughly double the amount of protein in the products with a protein claim than in the products without it (*p* < 0.01). Interestingly, products with a protein claim also had a significantly higher content of fiber (*p* < 0.05) than those without one. Regarding fat claims, there were no differences between total and saturated fat contents of products with or without fat claims. However, products with fat claims showed a significantly lower level of salt than those without. There was also no difference in fiber content between products that bore a fiber claim compared to those that did not. Products with a fiber claim did, however, exhibit a significantly higher energy and carbohydrate levels than those without (*p* < 0.01). There were no significant nutritional differences between products with an organic claim and those without. Nevertheless, products with a gluten-free claim were higher in saturates and protein, but lower in carbohydrates (*p* < 0.05).

### 3.3. Nutritional Comparison Between PBFSAs and Fish and Seafood Counterparts

**Figure 1:**
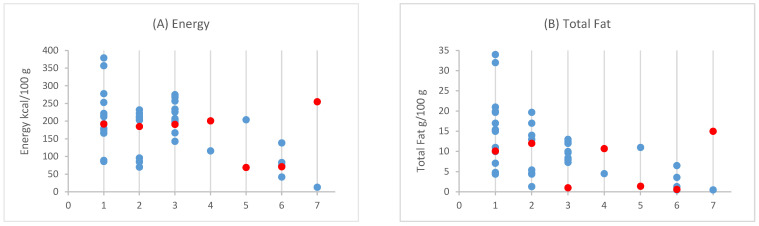

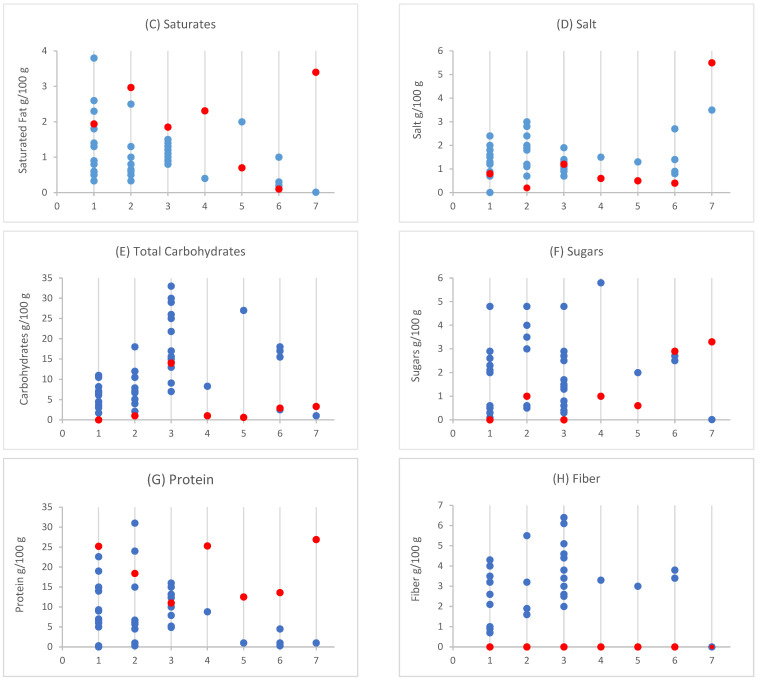
Values of (**A**) energy, (**B**) total fat, (**C**) saturates, (**D**) salt, (**E**) total carbohydrate, (**F**) sugars, (**G**) protein, and (**H**) fiber of PBFSAs (blue dots) and animal-based fish and sea products (red dots) [28]. For each plot, the 7 categories on the *x*-axis are expressed as follows: (1) tuna, (2) salmon, (3) cod, (4) mackerel, (5) squid, (6) shellfish/prawn, (7) roe/sturgeon caviar.

**Figure 2:**
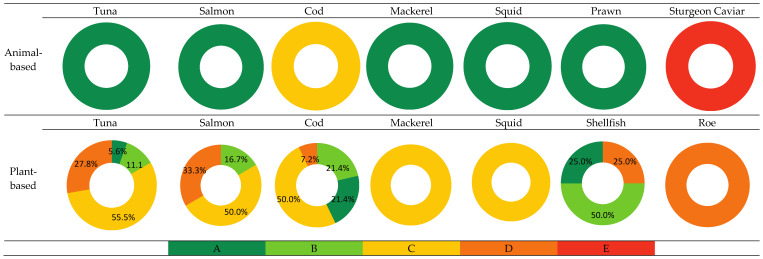
Nutri-Score (A–E) for categories of PBFSAs and animal-based counterparts. Animal-based categories include the following: tuna in oil (drained), fresh salmon, cod fish sticks, mackerel fillet in oil, frozen squid, prawns, and sturgeon caviar. Data are reported as percentages of products within a category that have received the same score. For animal-based and plant-based categories with a uniform color, the score is derived from a single product. Each color corresponds to a specific Nutri-Score.

The differences in terms of energy, nutrients, fiber, and salt between PBFSAs and their animal counterparts are presented in Figure 1. There was one product for each of the animal-based counterparts. Cod and squid analogs presented higher energy values compared to their animal product counterparts (i.e., cod fish sticks and frozen squid), while sturgeon caviar was nearly 19 times higher in calories than plant-based roe. As depicted in Figure 1, plant-based tuna, cod, squid, and shellfish exhibited a higher fat content compared to their corresponding animal-based products. However, plant-based mackerel and roe had lower fat levels than their animal-origin counterparts. Saturates were generally lower in all PBFSA categories compared to animal-based counterparts, except for plant-based squid and shellfish, which were higher in saturates. Regarding carbohydrates, all PBFSA categories had a higher carbohydrate content compared to their corresponding products of animal origin, except for plant-based roe, which had 1.0 g/100 g compared to 3.3 g/100 g in sturgeon caviar. Similarly, the sugar content tended to be higher in some PBFSAs. As shown in Figure 2, animal-based tuna and cod are naturally free of sugars, while the corresponding PBFSA tuna and cod recorded negligible sugar contents. The sugar content was higher in the animal-based prawns (2.9 g/100 g) and sturgeon caviar (3.3 g/100 g) in comparison to plant-based shellfish (2.5 (2.5–2.6) g/100 g) and roe (0 g/100 g). For fiber content, all PBFSA categories contained higher amounts of fiber than their animal counterparts, which contained no fiber, except for roe, where even the plant-based version did not contain fiber. In terms of protein, all PBFSA categories contained lower amounts of protein compared to their animal-based equivalents, except for cod which was similar in protein content (10.5 (7.9–13.0) g/100 g vs. 11.0 g/100 g). The largest differences in protein content between PBFSAs and animal products were noted in the mackerel and roe categories. Regarding salt content, only the PBFSA categories of cod (1.0 (1.0–1.1) g/100 g) and roe (3.5 g/100 g) had a lower salt content in comparison to their animal-based counterparts (1.2 g/100 g and 5.5 g/100 g, respectively).

### 3.4. Nutri-Score Comparison Between PBFSAs and Fish and Seafood Counterparts

The Nutri-Scores of the PBFSA categories and their animal-based counterparts are displayed in Figure 2. It is important to note that the Nutri-Scores of all of the animal product categories were based on one reference product, as previously clarified, hence they each received a single score. The same was applied to the plant-based mackerel, squid, and roe categories, being single items. Almost all of the animal-based foods had a Nutri-Score of A. The only exceptions were animal-based cod, which showed a score of C, and sturgeon caviar, which showed a score of E. These lower scores were mainly due to their high salt content, with salt penalties of 5 points for cod and 20 points for sturgeon caviar, exceeding those for other unfavorable Nutri-Score components. Sturgeon caviar was the only product among all plant-based and animal-based products to receive a score of E. It was also the only animal food to exhibit a score lower than its corresponding PBFSA, which had a D score. All of the products of the plant-based salmon category had lower Nutri-Scores than the animal-based salmon, with 16.7% having a score of B, 50% having a score of C, and 33.3% having a score of D. Some of the Nutri-Scores for PBFSAs matched those of their animal-based counterparts. These include 5.6% of plant-based tuna matching the score of A calculated for animal-based tuna, 25% of plant-based shellfish matching the score of A for animal-based prawn, and 50% of plant-based cod matching the score of C for animal-based cod. As a matter of fact, 42.8% of plant-based cod exhibited a higher score (A or B) than its animal-based counterpart.

## 4. Discussion

A shift towards sustainable and healthy dietary patterns is essential to ensure the optimal growth and wellbeing of present and future generations. These patterns typically involve a higher consumption of plant-based foods and a reduced consumption of animal-based foods. In this scenario, the food industry has recently put effort into the research and development of new plant-based alternatives, mainly targeting the structure, texture, sensory, and nutritional properties for various products, including not only seafood but also meat and dairy products [30,31,32]. These alternatives offer consumers more convenient options, as they are now more widely available at the stores consumers shop at and in formats consumers are familiar with cooking and eating, making it easier to increase their plant-based food intake [7].

Our study, which aimed to nutritionally evaluate fish analogs sold in Italy, found only 51 PBFSAs. This small number is consistent with the results of a recent study that assessed the nutritional quality of seafood alternatives in the global market [33]. The study retrieved 149 PBFSAs launched globally between 2002 and 2021 [33]. These findings highlight the lower availability of PBFSA products on the market in comparison to other plant-based alternatives, such as those of meat and dairy. Recent studies nutritionally evaluating plant-based alternatives retrieved 269 products of plant-based meat and sliced meat products, and 345 plant-based drinks replacing milk sold in Italian market [23,34], both being higher than the number of PBFSA products identified globally and in Italy.

Even though the quantity of PBFSA products seems small, their variety is on the rise, offering products like crab and fish cakes, fish burgers, tuna chunks and pate, calamari, caviar, fish fillets, fish fingers, fish sticks, shrimp, and even raw eel and tuna alternatives known as unami and ahimi, respectively [35]. In our study, the categorization of our products was fairly similar to that of the global market study [33], yet the latter included additional categories such as fish fingers, fish sticks, and filet, while ours included mackerel and cod. The formulation of such products usually relies on isolating proteins, polysaccharides, and phospholipids from plant-derived sources, including soy and peas, and transforming them into surimi-like gels [36]. The use of other products such as wheat, pseudocereals, rice, legumes, seeds, and nuts for the production of PBFSAs has also been recorded [33]. Although research shows that algae and microalgae are commonly used to manufacture PBFSAs due to their seafood-like flavor [16,37], in our study, only one product was formulated from algae.

Regarding the prevalence of products, tuna was the most common type of PBFSA, while in a global market study, plant-based shrimp was the most common [33]. There was also a considerable amount of caviar products listed in the global study while our study identified only one. This suggests that the distribution of the PBFSAs available in Italy is not similar to the distribution in the global market [33].

The nutritional analysis of these analogs revealed high variability in nutritional profiles. The variability may be attributed to the differences in the base ingredients of the products, as well as the method of preparation (e.g., products in oil, in sauces, breaded). Our results also revealed that PBFSAs generally contain higher levels of carbohydrates, fiber, and salt than their animal-based counterparts. These results align well with a previous study that compared the nutrient composition of fish and sea food with their plant-based alternatives launched in North America and Europe [38]. Such results are expected since cereals and pulses, which were used to formulate most of the products in our study, are high in carbohydrates compared to animal products. Also, research indicates that carbohydrates, such as starch, cellulose, and gums, are often used in the making of PBFSAs and other plant-based alternatives to provide thickening, gelling, emulsifying, or fluid-holding properties [37,39]. Animal-based cod having a higher carbohydrate content compared to other animal-based counterparts was likely due to the breading of the cod fish sticks, which were used as the animal-based counterpart. Despite that, the carbohydrate content of the fish sticks was still lower than that of the plant-based cod. The fiber content shown in our study aligns with a similar study conducted on meat products where all of the meat analogs presented a higher content of fiber than meat products [34]. This can be almost totally attributed to the cereal and legume formulations, which are high in fiber as well. Many PBFSAs also have a high salt content, which is consistent with findings from other studies evaluating the nutritional properties of plant-based fish/seafood and meat alternatives [34,38,40]. However, a study assessing the nutritional profile of seafood alternatives in the global market reported a variability in salt content among different products [33]. Salt is often added to these products for flavor enhancement, as well as for manipulating protein structure to increase their solubility and functionality [41]. The only exception in our study was with the animal-based sturgeon caviar, which had a higher content of salt compared to the plant-based counterpart. This is likely due to the high amount of salt used for the curing and storing of sturgeon caviar [42].

Regarding protein, almost all PBFSAs had lower levels compared to their conventional counterparts. These findings are similar to those observed in studies on plant-based dairy and meat alternatives, as well as studies on PBFSAs [23,33,34,38]. Animal-based sources of proteins generally hold a higher biological value than plant-based sources [43]. In addition, plant-based proteins can have reduced digestibility and bioavailability due to anti-nutritional factors like phytates and protease inhibitors [44]. Although previous research suggested that combining wheat and pea proteins improves the protein level and quality of plant-based products [33], most of the products in our study had lower protein levels than their animal-based counterparts despite being made of cereal and legume blends.

Our study noted a higher fat content in PBFSAs compared to their animal-based counterparts, except for mackerel and roe. The plant-based alternatives were also shown to be significantly higher in total fat when compared to animal-based fish and sea food in a recent US study [38]. However, these findings do not fully align with results from other studies on meat and dairy drink analogs [23,34]. For example, a study on meat analogs found a lower fat content in such products, particularly the steak analog, compared to animal-based meat [34]. Similarly, a study on plant-based drinks found that 97% of them had a lower fat content in comparison to regular milk [23]. The higher total fat content noted in mackerel could be due to the oil in the product, since “mackerel fillet in oil” was used as the animal-based counterpart. Sturgeon caviar is also recognized for its high fat content that contributes to its rich and melting texture, which is one of its main sensory qualities [45]. Although omega-3 PUFAs were not measured in our study, considering the well-documented health benefits associated with their intake and their abundant presence in certain fish [46,47], it is important that future studies assess the omega-3 PUFA profile in PBFSAs.

When it comes to nutrition claims, we found that products with nutrition and fat claims had lower salt levels, while products with a protein claim had significantly higher fiber levels. However, our results show that the presence of a claim on the product does not necessarily mean that the product has better nutritional quality, which is consistent with findings of previous study evaluating plant-based drinks sold in Italy [23]. The PBFSAs with a protein claim were indeed higher in protein content than those without one, aligning with the findings of Cutroneo et al. [34]. However, products with fat or fiber claims were not significantly different in terms of these nutrients from their counterparts without the claim. This is likely due to the high variability in the fat and fiber content, particularly in products not bearing such claims. As this may affect how consumers interpret nutritional information, it is crucial for consumers to understand that these claims do not indicate nor guarantee a tout court better nutritional quality of a product, as previously observed in literature [48,49,50,51,52]. Evidence has shown that such claims can create a ‘halo’ effect, influencing consumers to perceive products with claims as healthier, despite the lack of significant nutritional differences [53].

Additionally, very few studies nutritionally assessed plant-based alternatives and even fewer attempted to score the products according to their nutritional quality. Because of that, a thorough and clear comparison was definitely a challenge. The Nutri-Score analysis revealed that most animal-based products scored even better than plant-based ones. Interestingly, a study on plant-based meat found the opposite, showing that a higher number of plant-based meat products presented better Nutri-Scores than meat-based ones [34]. Our results can be mainly attributed to the higher salt content in PBFSAs, as salt carries a higher penalty (up to 20 points) in Nutri-Score than other unfavorable components. While protein and fiber content contributed to the score positively, the negative impact of the higher salt content was greater, leading to a more negative Nutri-Score. Despite the usefulness of the Nutri-Score, this may be viewed as a limitation of this labeling system, as it may influence the comparison between PBFSAs and animal-based products and may push consumers to view PBFSAs with lower Nutri-Scores as less healthy, especially when comparing their scores to those of their animal-based counterparts. Therefore, it is recommended that the food industry both enhances the protein quality and reduces the salt content in PBFSA products to improve their nutritional characteristics and scores. It is also important to note that for all animal-based categories and some plant-based categories, namely mackerel, squid, and roe, the score was derived from a single product. For other PBFSA categories, the score for the categories was derived from multiple products. This may result in inaccurate conclusions due to the high difference in number of products within the categories compared.

It is crucial to consider both the limitations and the strengths of our study. One limitation is the relatively small number of products included and the variability of products across the different categories. As mentioned above, only one product was considered for each category of the animal-based counterparts, as well as for some plant-based categories (mackerel, squid, and roe). This, combined with our study’s focus on products sold specifically in Italy, may impact the robustness of the findings by limiting the generalizability of our results and may not fully represent the variety of products found in other countries. The heterogeneity noted in the recipes of the plant-based products (e.g., products in oil, in sauces, breaded, etc.) made the comparison complex. Additionally, finding an animal-based counterpart that matched all PBFSA products in each category was challenging. Although animal-based counterparts were chosen to align with the most common PBFSA products in each category, this may have influenced the results and made it difficult to compare and draw concrete conclusions. Another limitation is that this study only evaluated the mandatory nutritional information reported on the product nutrition labels according to the European Council Regulations. Because of that, other nutritional components, such as micronutrients and long-chain fatty acids that are usually associated with the consumption of fish and seafood, were not evaluated. Considering the strengths, to the best of our knowledge, this is the first study to evaluate the nutritional properties of PBFSAs sold in the Italian market. Although the number of products retrieved may seem low, our study considered a wide range of supermarkets in Italy and is believed to have covered the majority of PBFSAs currently available on the market. We also considered in our nutritional evaluation the Nutri-Score of the products and were able to assess the presence of nutrition claims, organic declarations, and gluten-free indications.

## 5. Conclusions

Our study found that PBFSAs in Italy nutritionally differed from their animal-based counterparts, as they were higher in levels of carbohydrates, fiber, and salt, but lower in protein. Therefore, it is essential for consumers to understand that PBFSAs currently do not equate fish and seafood from a nutritional point of view and should not completely substitute neither animal-based nor plant-based foods. Instead, they can play a great role in diversifying and complimenting plant-based diets. Our findings also emphasized the importance of properly understanding nutrition claims or front-of-pack nutrition labels such as the Nutri-Score, as they are not necessarily an indicator of the overall nutritional quality. There is significant room for improvement in terms of food concept and the implementation of nutritional characteristics for plant-based alternatives. The number of products included in the study and their variability highlight the need for more comprehensive research to better understand the nutritional profile of PBFSAs sold in the Italian market, with future studies focusing on strategies such as increasing product diversity and addressing underrepresented categories. Future studies should also focus on evaluating other nutritional aspects, such as micronutrients and long-chain omega-3 fatty acids. The evaluation of the sustainability of these products is also needed, considering not only the nutritional and health factors, but also environmental and social ones, such as consumer perceptions and preferences. Exploring these factors will aid in assessing their viability as part of an Italian sustainable diet. Finally, these results may be useful for the food industry in developing plant-based products with high sensory quality and nutritional properties for consumers who seek fish alternatives.

## Figures and Tables

**Table 1:** Number and characteristics of PBFSAs.

		Number (n)
	Total PBFSAs	51
Categories	Tuna	18
	Salmon	12
	Cod	14
	Mackerel	1
	Squid	1
	Shellfish	4
	Roe	1
Nutrition Claim	Yes	26
	No	25
Protein-related Claim	Yes	23
	No	28
Fat-related Claim	Yes	22
	No	29
Fiber-related Claim	Yes	5
	No	46
Organic Declaration	Yes	4
	No	47
Gluten-free Indication	Yes	5
	No	46
Formulation (main ingredient)	Cereals	10
	Legumes	5
	Algae	1
	Mixture ^1^	35

^1^ Items were mainly formulated from a mixture of cereals and legumes.

**Table 2:** Nutritional composition of plant-based fish and seafood analogs.

	Energy		Total Fat	Saturates	Total Carbohydrates	Sugars	Fiber	Protein	Salt
	kcal/100 g	kJ/100 g	g/100 g	g/100 g	g/100 g	g/100 g	g/100 g	g/100 g	g/100 g
Tuna (*n* = 18)	193 (91–213) ^ab^	800 (712–917) ^ab^	15.0 (10.0–19.7) ^a^	1.3 (0.6–1.8) ^a^	6.2 (4.4–6.7) ^b^	0.6 (0.3–2.3) ^a^	1.6 (0–3.2) ^a^	9.0 (6.1–14.0) ^ab^	1.3 (1.2–1.8) ^ab^
Salmon (*n* = 12)	211 (91–213) ^ab^	877 (381–882) ^ab^	13.5 (5.0–17.0) ^ab^	0.6 (0.6–1.2) ^a^	6.7 (5.9–9.2) ^b^	0.6 (0.5–3.3) ^a^	1.8 (0–3.2) ^a^	6.7 (5.1–10.9) ^ab^	1.8 (1.5–2.2) ^a^
Cod (*n* = 14)	217 (201–257) ^a^	904 (838–1073) ^a^	10.0 (8.4–12.0) ^ab^	1.1 (1.0–1.3) ^a^	19.4 (14.2–26.0) ^a^	1.4 (0.4–2.5) ^a^	3.6 (2.5–4.6) ^a^	10.5 (7.9–13.0) ^a^	1.0 (1.0–1.1) ^b^
Mackerel (*n* = 1)	116 ^ab^	485 ^ab^	4.5 ^ab^	0.4 ^a^	8.3 ^ab^	5.8 ^a^	3.3 ^a^	8.8 ^ab^	1.5 ^ab^
Squid (*n* = 1)	204 ^ab^	854 ^ab^	11.0 ^ab^	2.0 ^a^	27.0 ^ab^	2.0 ^a^	3.0 ^a^	1.0 ^ab^	1.3 ^ab^
Shellfish (*n* = 4)	79 (59–111) ^b^	331 (245–464) ^b^	2.5 (0.9–5.1) ^b^	0.3 (0.2–0.7) ^a^	16.3 (9.0–17.5) ^ab^	2.5 (2.5–2.6) ^a^	3.4 (1.7–3.6) ^a^	0.7 (0.3–2.8) ^b^	1.2 (0.9–2.1) ^ab^
Roe (*n* = 1)	13 ^ab^	54 ^ab^	0.5 ^ab^	0.0 ^a^	1.0 ^ab^	0.0 ^a^	0.0 ^a^	1.0 ^ab^	3.5 ^ab^

Data are presented as median (25th percentile–75th percentile). Different letters within the same column indicate. significant difference at *p* < 0.05 among the different PBFSA categories. (Kruskal–Wallis test for independent samples and multiple pairwise comparison test).

**Table 3:** Number of items and nutritional composition of PBFSAs with nutrition claims including fat, protein, fiber, organic declaration, and gluten-free indication.

	Energy		Total Fat	Saturates	Total Carbohydrates	Sugars	Fiber	Protein	Salt
	kcal/100 g	kJ/100 g	g/100 g	g/100 g	g/100 g	g/100 g	g/100 g	g/100 g	g/100 g
Nutrition Claim									
Yes	205 (168–232)	857 (700–979)	10.0 (7.9–12.4)	1.0 (0.8–1.4)	9.4 (4.5–17.0)	1.1 (0.5–2.7)	2.6 (1.9–4.0)	10.0 (6.7–14.0) *	1.1 (0.9–1.3) *
No	203 (139–213)	850 (580–882)	11.0 (6.5–17.0)	0.9 (0.5–1.3)	6.7 (6.3–13.0)	2.0 (0.5–2.5)	2.0 (0.0– 3.3)	6.1 (2.8–9.1)	1.8 (1.3–2.0)
Protein Claim									
Yes	207 (176–257)	864 (712–1073)	10.0 (7.9–12.1)	1.0 (0.8–1.4)	11.0 (4.4–21.8)	1.3 (0.5–2.5)	2.6 (2.0–4.3) *	11.0 (7.0–14.0) *	1.2 (1.0–1.5)
No	200 (101–213)	836 (423–882)	12.0 (4.5–17.0)	0.8 (0.5–1.3)	6.9 (6.5–11.3)	1.3 (0.5–2.6)	1.5 (0.0–3.3)	6.1 (1.0–8.8)	1.5 (1.1–2.0)
Fat Claim									
Yes	206 (167–257)	861 (697–1073)	10.0 (7.9–13.0)	1.0 (0.65–1.40)	9.2 (4.5–15.6)	1.1 (0.5–2.3)	2.8 (2.0–4.0)	10.0 (6.7–14.0)	1.1 (0.9–1.3) *
No	203 (143–222)	850.0 (594–882)	10.0 (6.5–17.0)	1.0 (0.6–1.3)	7.10 (6.3–15.5)	2.0 (0.5–2.6)	2.0 (0.0–3.3)	6.4 (4.7–11.2)	1.5 (1.3–2.0)
Fiber Claim									
Yes	268 (226–271) *	1125.0 (945–1134) *	12.0 (12.0–12.1)	1.20 (1.0–1.3)	29.0 (15.6–30.0) *	2.7 (1.4–2.9)	3.0 (2.6–3.4)	10.0 (10.0–12.3)	1.1 (1.0–1.1)
No	199 (139–213)	830.0 (580–882)	10.0 (5.4–17.0)	1.0 (0.6–1.4)	6.85 (4.5–13.0)	0.6 (0.5–2.5)	2.6 (0.0–3.4)	6.7 (5.0–14.0)	1.3 (1.0–1.8)
Organic Declaration									
Yes	210 (160–287)	878 (668–1200)	14.7 (9.3–23.7)	2.6 (1.5–2.6)	5.2 (3.8–7.3)	2.7 (1.5–4.4)	1.7 (0.0–3.4)	12.1 (9.1–19.5)	1.4 (0.7–1.7)
No	203 (143–222)	850 (594–917)	10.0 (6.5–15.0)	1.0 (0.6–1.3)	7.9 (6.1–17.0)	0.8 (0.5–2.5)	2.6 (0.0–3.4)	6.7 (5.0–13.0)	1.3 (1.0–1.8)
Gluten-Free Indication									
Yes	216 (203–357)	906 (850–1494)	15.4 (14.0–32.0)	2.6 (2.5–2.6) *	4.0 (3.6–6.3) *	2.3 (2.0–3.0)	2.1 (0.0–3.3)	14.9 (9.3–15.0) *	1.3 (0.9–1.5)
No	202 (143–222)	844 (594–917)	10.0 (6.5–15.0)	1.0 (6.0–1.3)	8.1 (6.6–17.0)	0.7 (0.5–2.5)	2.6 (0.0–3.4)	6.7 (5.0–13.0)	1.3 (1.0–1.8)

Data are presented as median (25th percentile–75th percentile). * Asterisks indicate significant differences at *p* < 0.05 between items with claims, declarations, and indications versus those without (Mann–Whitney test for independent samples).

## Data Availability

The original contributions presented in this study are included in the article/Supplementary Materials. Further inquiries can be directed to the corresponding author.

## Supplementary Materials

The following supporting information can be downloaded at: https://www.mdpi.com/article/10.3390/foods14030394/s1, Table S1: Nutritional composition of animal-based fish and seafood.
