# Supplementary material for: Nutritional Quality of Plant-Based Fish and Seafood Analogs: A Study of the Italian Market
Source: Foods. 2025 Jan 25;14(3):394. doi: 10.3390/foods14030394 (PMC11816721; doi:10.3390/foods14030394)
Supplement: Supplementary file 1 [file foods-14-00394-s001.zip › foods-3417319-supplementary.pdf]

Table S1 Nutritional Composition of Animal-Based Fish and Seafood

	Energy		Total Fat	Saturates	Total Carbohydrates	Sugars	Fiber	Protein	Salt*
	**kcal/100 g**	**kJ/100 g**	**g/100 g**	**g/100 g**	**g/100 g**	**g/100 g**	**g/100 g**	**g/100 g**	**g/100 g**
**Tuna in Oil (Drained)**	192	802	10.1	1.9	0.0	0.0	0.0	25.2	0.8
**Fresh Salmon**	185	773	12.0	3.0	1.0	1.0	0.0	18.4	0.2
**Cod Fish Sticks**	191	801	10.1	1.9	14.0	0.0	0.0	11.0	1.2
**Mackerel Fillet in Oil**	201	842	10.7	2.3	1.0	1.0	0.0	25.3	0.6
**Frozen Squid**	69	291	1.4	0.7	0.6	0.6	0.0	12.5	0.5
**Prawns**	71	300	0.6	0.1	2.9	2.9	0.0	13.6	0.4
**Sturgeon Caviar**	255	1065	15.0	3.4	3.3	3.3	0.0	26.9	5.5

Nutritional data is collected from the Food Composition Database for Epidemiological Studies in Italy (BDA) [21]. * Salt levels were calculated by multiplying sodium levels (mg/100g) by 2.5.
